# Combined effects of global climate change and nutrient enrichment on the physiology of three temperate maerl species

**DOI:** 10.1002/ece3.5802

**Published:** 2019-12-05

**Authors:** Zujaila Nohemy Qui-Minet, Jérôme Coudret, Dominique Davoult, Jacques Grall, Miguel Mendez‐Sandin, Thierry Cariou, Sophie Martin

**Affiliations:** ^1^ Sorbonne Universités CNRS UMR 7144 Adaptation et Diversité en Milieu Marin Station Biologique de Roscoff Roscoff France; ^2^ Université de Bretagne Occidentale IUEM Plouzané France; ^3^ Sorbonne Universités CNRS, FR2424 Station Biologique de Roscoff Roscoff France

**Keywords:** calcification, maerl, nitrate, ocean acidification, ocean warming, phosphate, photosynthesis, respiration

## Abstract

Made up of calcareous coralline algae, maerl beds play a major role as ecosystem engineers in coastal areas throughout the world. They undergo strong anthropogenic pressures, which may threaten their survival. The aim of this study was to gain insight into the future of maerl beds in the context of global and local changes. We examined the effects of rising temperatures (+3°C) and ocean acidification (−0.3 pH units) according to temperature and pH projections (i.e., the RCP 8.5 scenario), and nutrient (N and P) availability on three temperate maerl species (*Lithothamnion corallioides*, *Phymatolithon calcareum*, and *Lithophyllum incrustans*) in the laboratory in winter and summer conditions. Physiological rates of primary production, respiration, and calcification were measured on all three species in each treatment and season. The physiological response of maerl to global climate change was species‐specific and influenced by seawater nutrient concentrations. Future temperature–pH scenario enhanced maximal gross primary production rates in *P. calcareum* in winter and in *L. corallioides* in both seasons. Nevertheless, both species suffered an impairment of light harvesting and photoprotective mechanisms in winter. Calcification rates at ambient light intensity were negatively affected by the future temperature–pH scenario in winter, with net dissolution observed in the dark in *L. corallioides* and *P. calcareum* under low nutrient concentrations. Nutrient enrichment avoided dissolution under future scenarios in winter and had a positive effect on *L. incrustans* calcification rate in the dark in summer. In winter conditions, maximal calcification rates were enhanced by the future temperature–pH scenario on the three species, but *P. calcareum* suffered inhibition at high irradiances. In summer conditions, the maximal calcification rate dropped in *L. corallioides* under the future global climate change scenario, with a potential negative impact on CaCO_3_ budget for maerl beds in the Bay of Brest where this species is dominant. Our results highlight how local changes in nutrient availability or irradiance levels impact the response of maerl species to global climate change and thus point out how it is important to consider other abiotic parameters in order to develop management policies capable to increase the resilience of maerl beds under the future global climate change scenario.

## Graphical abstract

Maerl beds play a major role as ecosystem engineers in coastal areas. We examined the effects of rising temperatures (+3°C) and ocean acidification (−0.3 pH units) according to temperature projections (i.e. the RCP8.5 scenario), and nutrient (N and P) availability on three temperate maerl species. Responses were species‐specific and varied with the season, irradiance and nutrient availability.

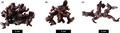

## 1 INTRODUCTION

The coastal system is under severe threat and is considered as one of the most vulnerable environments due to the strong influence of global and local anthropogenic pressures. Global climate change is expected to affect abundance, diversity, and productivity of marine populations (Barange & Harris, 2003), thus becoming a major driver of the future state of marine ecosystems (Duarte et al., 2013). Atmospheric carbon dioxide (CO_2_) concentrations have risen from 278 parts per million (ppm) at the start of the Industrial Revolution to the current level of 410 ppm. Approximately, one‐third of CO_2_ emissions are absorbed by the ocean and affect its chemistry and physics (Gattuso et al., 2015), causing a global average increase in temperature of 0.8°C and a decline in pH of 0.1 units of the surface oceans since the industrial revolution, phenomena known as ocean warming and ocean acidification (OA), respectively (Gattuso et al., 2015). Marine coastal ecosystems are also influenced by other, more local anthropogenic pressures, which are growing due to various human activities in coastal areas. Considering this, coastal eutrophication is attributed to the enrichment of nutrients such as phosphates and nitrates resulting from agricultural runoff or wastewater discharged into the sea via rivers (Diaz‐Pulido & McCook, 2008; Duarte et al., 2013; Russel & Connell, 2009; Salisbury, Green, Hunt, & Campbell, 2008; Strong, Kroeker, Teneva, Mease, & Kelly, 2014).

Global climate change is expected to alter dominance relationships among primary producers, in particular between calcifying and noncalcifying macroalgal species (Brodie et al., 2014; Celis‐Plá et al., 2015; Falkenberg, Connell, & Russel, 2013). However, these changes will depend on local factors such as nutrient availability (Celis‐Plá et al., 2015; Fabry, Seibel, Feely, & Orr, 2008). Red calcareous coralline macroalgae are among the most sensitive organisms to OA (Martin & Hall‐Spencer, 2017) because they precipitate high‐magnesium calcite, which is the most soluble form of biogenic calcium carbonate (CaCO_3_) (Morse, Andersson, & Mackenzie, 2006). Among them, free‐living nongeniculate red coralline algae, called rhodoliths or maerl, are distributed in coastal ecosystems throughout the world (Foster, 2001) and experience different levels of pH and temperature changes depending on their location (Qui‐Minet et al., 2018). Previous studies on maerl and other red coralline algae have given insights into their individual responses to OA and warming, which are useful for a better understanding of how species physiology responds to these factors alone or in combination. Although the responses of coralline algae are species‐specific (Martin, Charnoz, & Gattuso, 2013; Noisette, Egilsdottir, Davoult, & Martin, 2013; Vazquez‐Elizondo & Enriquez, 2016), most studies have revealed adverse effects of the combination of ocean acidification and warming on coralline algal physiology (Martin & Hall‐Spencer, 2017).

Owing that productivity is limited by nutrient availability, moderate nutrient enrichment can benefit algae by reducing nutrient limitation and allowing them to cope with the metabolic cost of adapting to global climate change (Celis‐Plá et al., 2015; Hofmann, Heiden, Bischof, & Teichberg, 2014). However, nutrient enrichment can threaten maerl survival and favor the development of fleshy epiphytic macroalgae (Grall & Hall‐Spencer, 2003; Steller, Riosmena‐Rodrίguez, Foster, & Roberts, 2003). Research focusing on the effect of nutrient enrichment under current conditions of pH and temperature in coralline algae has reported that increased nitrate concentrations do not affect photosynthesis and calcification (Belliveau & Paul, 2002; Bjork, Mohammed, Bjorklund, & Semesi, 1995). However, phosphates have been described as “crystal poison” since they have shown to act as an inhibitor of calcification and growth of coralline algae (Bjork et al., 1995; Kinsey & Davies, 1979; Simkiss, 1964). Studies specifically addressing the interaction of global climate change with nutrient enrichment on the physiology of coralline algae are scarce, and none have explored the response of maerl species.

**Figure 1:**
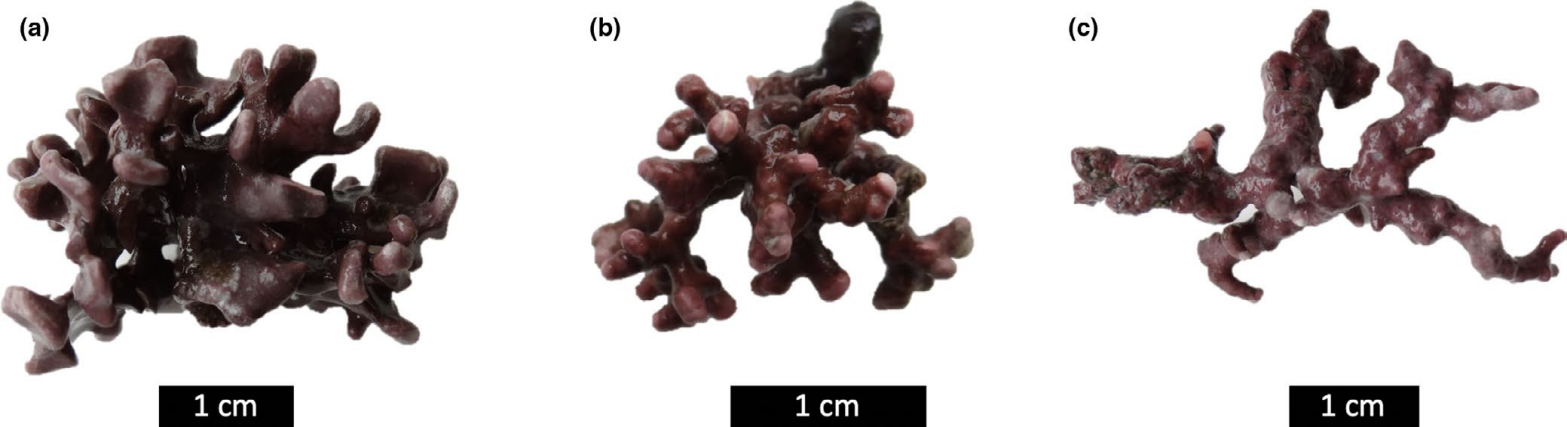
Specimens of (a) *Lithothamnion corallioides*, (b) *Phymatolithon calcareum*, and (c) *Lithophyllum incrustans* collected in the Roz maerl bed in the Bay of Brest (Brittany, France) (photos Coralie Delaunay). Scale bars = 1 cm

Maerl beds in the Bay of Brest (Brittany, France) are highly productive benthic systems (Martin, Clavier, Chavaud, & Thouzeau, 2007) subject to seasonal variations in physicochemical parameters (Salt et al., 2015, Qui‐Minet et al., 2018). In the present study, we selected three maerl species co‐occurring in the Bay of Brest: *Lithothamnion corallioides* (P. Crouan & H. Crouan) P. Crouan & H. Crouan, *Phymatolithon calcareum* (Pallas) W.H. Adey & D.L. McKibbin ex Woelkering & L.M. Irvine, and *Lithophyllum incrustans* Philippi. These species are relevant because of the ecological role they play in the northeastern Atlantic. They are the main maerl species found in France, and *L. corallioides* and *P. calcareum* are also the main species forming maerl beds in Europe. Although all three species were collected at the same location and acclimated to the same conditions, their geographical distribution is not the same, with the northern limits of *P. calcareum* being found all the way to southern Norway, whereas the northern distribution of *L. corallioides* and *L. incrustans* stops in Ireland. Further, *L. incrustans* observes a more southern distribution (Hernández‐Kantún et al., 2016), being present in the Caribbean (BIOMAERL, 1998; Hernández‐Kantún et al., 2015; McCoy & Kamenos, 2015) and found in rock pool habitats subject to high variability in physicochemical parameters and exposed to high temperatures and low pH values at low tide (Egilsdottir, Noisette, Noël, Olafsson, & Martin, 2013; Legrand et al., 2018; Williamson et al., 2014). This species is rarely found below 8 m depth (Ford, Hardy, & Edyvean, 1983), whereas *L. corallioides* and *P. calcareum* are found in shallow environments, but also at depths up to 20 and 30 m, respectively (Birkett, Maggs, & Dring, 1998). In Brittany, *L. corallioides* and *P. calcareum* are the most abundant species, whereas *L. incrustans* is present at lower abundances (Grall & Hall‐Spencer, 2003). In the Bay of Brest, maerl beds are mainly formed by *L. corallioides* (Grall, 2002; Qui‐Minet et al., 2018). In this bay, one maerl bed (Roz) presents all the three maerl species *L. corallioides*, *P. calcareum*, and *L. incrustans* together (Figure 1).

The purpose of this study was to test (a) the individual and combined effects of global climate change (ocean warming and acidification) and local nutrient loading on various species of maerl and (b) if different species coming from the same location respond similarly due to convergent adaptation. We considered present temperature–pH conditions and future temperature–pH conditions projected for 2,100 according to the representative concentration pathway RCP 8.5 global change scenario (Gattuso et al., 2015) as well as enrichment in nitrate (NO_3_^−^) and phosphate (PO_4_^3−^), the main nutrients originating from human activities affecting the Bay of Brest (Le Pape & Menesguen, 1997). To consider the potential seasonal variability of the response of maerl to OA and warming (Martin, Cohu, Vignot, Zimmerman, & Gattuso, 2013; Martin & Gattuso, 2009), we carried out our study under winter and summer conditions. We hypothesized that (a) the future scenario of ocean warming and acidification will negatively affect calcification in the three maerl species; (b) nutrient enrichment will exacerbate the impact of global climate change on the three species; and (c) although the three maerl species coexist in the same location and hence are adapted to the same environmental conditions, species‐specific responses occur due to divergent ecological traits such as morphology and pigment concentrations.

## 2 MATERIALS AND METHODS

### 2.1 Collection site

The three maerl species, *L. corallioides*, *P. calcareum*, and *L. incrustans* (Figure 1), were collected in the Roz maerl bed (48°19′58″N, 04°19′57″W), located in the southern basin of the Bay of Brest (Brittany, France), where it covers a surface of 1.4 km^2^ (Qui‐Minet et al., 2018). The three here studied species are present in different abundances, being *L. corallioides* the most abundant and the only one distributed along the Bay of Brest.

Temperature and pH in the Bay of Brest average 10°C and 8.04 in the winter, and 18°C and 8.00 in the summer, respectively. Local nutrient conditions are strongly variable according to the season as reported by Le Pape and Menesguen (1997), and Qui‐Minet et al. (2018); nitrate concentrations are significantly higher in winter (±30 μmol/L) relative to summer (<1 μmol/L) while phosphate concentrations remain below 1 μmol/L during both summer and winter seasons. Incident irradiance in the Bay of Brest varies with tides. In the collection site, it can vary from <1 to 320 μmol photons m^−2^ s^−1^ in winter and from 1 to 645 μmol photons m^−2^ s^−1^ in the summer (Qui‐Minet et al., 2018). The Roz bed is located at 0.7 m depth (chart datum), with maximal water depth amplitudes of 8 m (Daniel, 1995).

Physicochemical parameters also vary on a daily basis, in the summer in the Roz bed, temperature and pH can observe a mean variation of 0.7°C and 0.08 pH units, respectively (Qui‐Minet et al., 2018).

### 2.2 Biological material

Living specimens of the three species were collected with a 0.1 m^2^ Van Veen grab in January 2016 (winter experiment) and in June 2016 (summer experiment). The algae were brought to the laboratory in a box filled with seawater (seawater temperature did not differ much from air temperature). After cleaning, thalli were randomly distributed into the aquaria of the experimental system and kept under in situ conditions of temperature and pH. In each aquarium, there were two sets of 5–7 g dry weight (DW) of *L. corallioides*, 5–7 g (DW) of *P. calcareum*, and 7–9 g (DW) of *L. incrustans* attached with a nylon wire and labeled with small plastic numbers and additional unlabeled thalli (approximately 10 g per species).

### 2.3 Experimental conditions and setup

**Figure 2:**
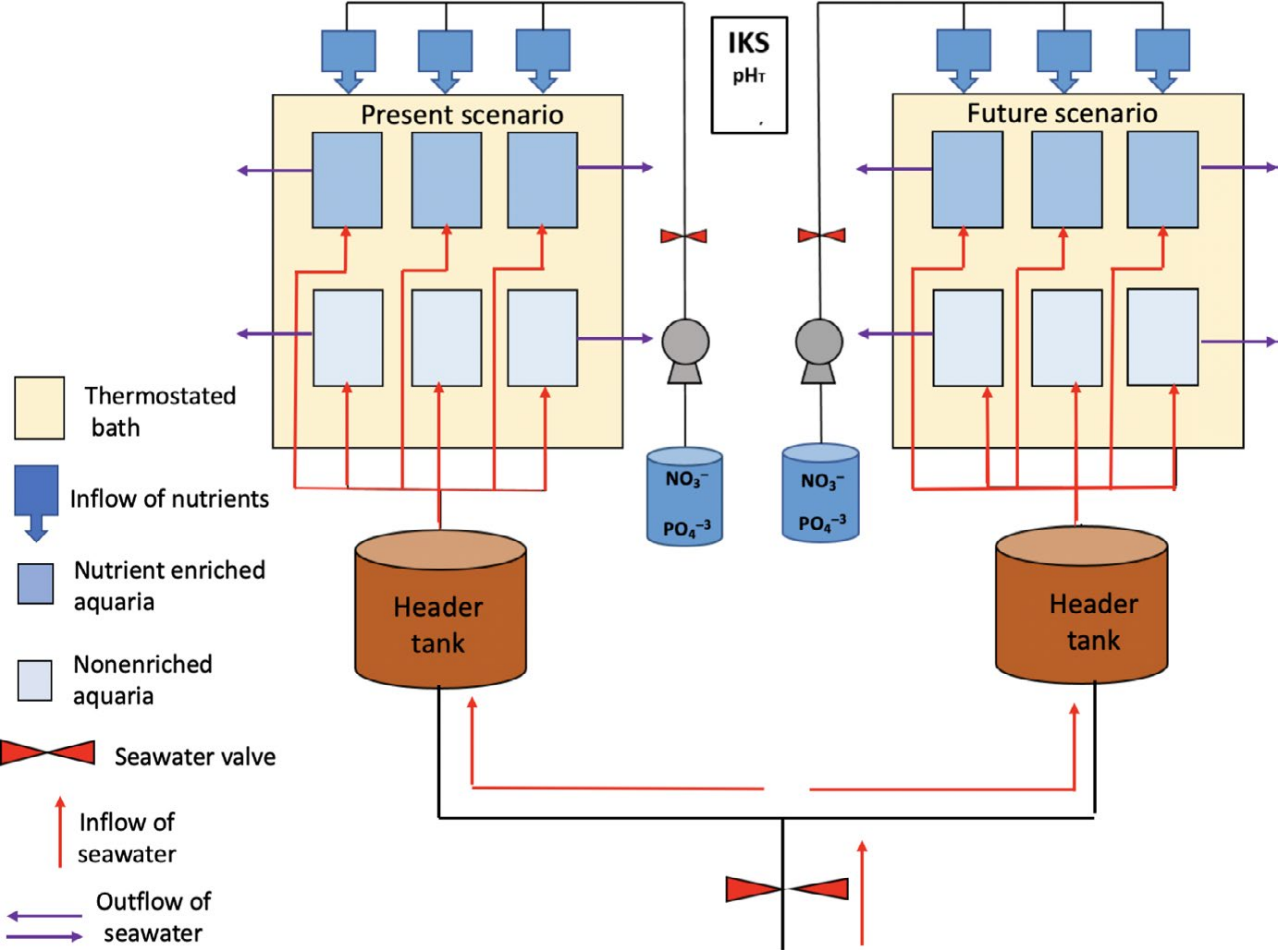
Schematic diagram of the experimental laboratory setup with two scenarios: present pH and temperature and future (RPC 8.5) projected pH and temperature by 2,100. All aquaria were maintained at constant temperature in a thermostated bath. Each aquarium contained 2 sets of maerl thalli for each species

Two experiments were conducted for both winter (January–April 2016) and summer (June–September 2016) conditions. For each season, two scenarios of temperature and pH were chosen: a present scenario and a future scenario. Each temperature–pH test scenario was crossed with nutrient availability conditions, resulting in four different treatments: (a) present scenario‐low nutrients concentrations (Pr‐LN); (b) present scenario‐high nutrients concentrations (Pr‐HN); (c) future scenario‐low nutrients concentrations (Fu‐LN); and (d) future scenario‐high nutrients concentrations (Fu‐HN; Figure 2).

For the tested present temperature–pH conditions, temperature and pH were determined from mean values measured in situ in winter and summer (temperature: 10 and 18°C, respectively; pH on the total scale (pH_T_): 8.04 and 8.00, respectively) (Qui‐Minet et al., 2018). Acclimation to the experimental system under controlled conditions lasted 3 weeks. For the tested projected future temperature–pH conditions, values were selected according to the future RCP 8.5 scenario (+2.7°C and −0.33 pH units; Gattuso et al., 2015). Temperature and pH were progressively increased and decreased by ~0.4°C and ~0.05 pH units, respectively, every 2 days during 2 weeks. An experimental thermostated system was set up for both temperature–pH scenarios (present and future). Each experimental system consisted of a thermostated bath containing six aquaria (10 L), three with nutrient enrichment and three without nutrient enrichment (12 aquaria in total; Figure 2). Aquaria were supplied at a rate of 9 L/hr from a 100 L header tank continuously supplied with filtered seawater pumped in front of the Roscoff Marine Biological Station (*Station Biologique de Roscoff*). Due to constraining space conditions, only one header tank was used per scenario. However, this was an open‐flow system with filtered seawater (5 µm) and water renewal rate (~54 L/hr) with high cleaning frequency (weekly) in the header tank to prevent any contamination event and tank effect. Nutrients were distributed continuously from a peristaltic pump to increase the concentrations of PO_4_^3−^ and NO_3_^−^ of 1 and 40 μmol/L, respectively, the mean values measured in the Bay of Brest (Qui‐Minet et al., 2018). Concentrations of silicic acid (Si(OH)_4_^−^) were not controlled under either scenario and ranged from 1.1 to 2.8 μmol/L in winter and from 1.67 to 2.65 μmol/L in summer. Temperature inside the aquaria was adjusted with two cooling elements, one that controlled the temperature inside the 100 L header tank and another one that maintained the desired temperature inside the thermostated water bath. pH was monitored and controlled by an off‐line feedback system (IKS Aquastar) that regulated the addition of CO_2_ in the 100 L header tanks Ambient light was kept at 30 μmol photons m^−2^ s^−1^ with a light/dark photoperiod of 10/14 hr in winter and 14/10 hr in summer.

### 2.4 Monitoring of the seawater parameters

Temperature and pH were measured on a daily basis; total alkalinity (T_A_) was measured every week, and nutrients were measured every 2 weeks. The pH values of the system were adjusted based on daily measurements of pH_T_ and temperature in the 12 aquaria using a pH meter (HQ40D, Hach Lange, Ltd portable LDO™) calibrated with Tris/HCl and 2‐aminopyridine/HCl buffers (Dickson, Sabine, & Christian, 2007). Samples for nutrient assays and T_A_ analyses were collected from filtered water using 0.22 μm Sterivex cartridges (Millipore). Samples for NO_3_^−^, PO_4_^3−^, and Si(OH)_4_ analyses were frozen at −20°C until analysis, and ammonium (NH_4_^+^) samples were stored in 100 ml borosilicate glass bottles. Samples were treated with mercuric chloride (0.02% v/v; Dickson, Afghan, & Anderson, 2003) and stored in a dark, cool place. Analysis of NO_3_^−^, PO_4_^3−^, and Si(OH)_4_ concentrations were done by spectrophotometry using a Technicon autoanalyzer according to Aminot and Kérouel (2007). NH_4_^+^ concentrations were determined using the Solorzano (1969) method. T_A_ was measured by 0.01 N HCl potentiometric titration on an automatic titrator (Titroline alpha, Schott Si Analytics) calibrated on the National Bureau of Standards scale and by using the Gran method of nonlinear least‐squares fit applied to pH variations from 3.5 to 3.0 mEq/L (Dickson et al., 2007). The T_A_ measurements were corrected using standards provided by A.G. Dickson (Batch 111) and had a reproducibility of ±4 μmol/kg. Dissolved inorganic carbon (DIC), *p*CO_2_, calcite (Ω_Ca_), and aragonite (Ω_Ar_) were calculated from triplicates values of temperature, pH_T_, salinity, pressure, silicate, and phosphate concentrations, by using the software CO_2Sys_, Excel Macro version 2.1 (originally designed by Lewis & Wallace, 1998). Calculations were based on a set of constants K1 and K2 from Mehrbach, Culberson, Hawley, & Pytkowicz (1973) refitted by Dickson & Millero (1987).

### 2.5 Metabolic rate measurements

Metabolic measurements were performed after 3 months of culture in each treatment. Net primary production (NPP) and net calcification rates in light (G_L_) were measured at an ambient irradiance of 30 μmol photons m^−2^ s^−1^. Respiration (R) and net calcification rates in the dark (G_D_) were measured in dark at the beginning or at the end of the day. Net primary production (NPP) and net calcification (G) versus irradiance (E) curves were defined for the three maerl species only in the nonenriched nutrient condition (Pr‐LN and Fu‐LN) in winter (under irradiances of 0, 30, 50, 100, 200, and 400 μmol photons m^−2^ s^−1^) and in summer (under irradiances of 0, 30, 70, 150, 320, and 600 μmol m^−2^ s^−1^) conditions. NPP and G versus E curves in the nutrient‐enriched conditions (Pr‐HN and Fu‐HN) were only performed for *L. corallioides* in summer. Incubations were done in 185 ml acrylic respirometry chambers (Engineering & Design Plastics Ltd). Chambers contained a plastic grid above a stir bar (100 rpm) which ensured water homogeneity. Control incubations without algae were carried out to correct fluxes from biological activity in seawater. Incubations lasted between 1 and 2.5 hr to maintain oxygen saturation above 80%, and pH changes lower than 0.1 pH units during incubation. NPP and R were estimated by measuring oxygen concentrations at the beginning and at the end of incubation with a noninvasive optical fiber system (FIBOX 3, PreSens). Reactive oxygen spots of the chambers were calibrated before the incubations with 0% and 100% oxygen buffers. Calcification rates were estimated using the alkalinity anomaly technique (Smith & Key, 1975), which is a good estimator for short‐term incubations. It is based on a decrease of two moles total alkalinity (T_A_) by two equivalents per molecule of CaCO_3_ precipitated (Wolf‐Gladrow, Zeebe, Klaas, Kortzinger, & Dickson, 2007). Seawater was sampled at the beginning and at the end of incubation, and analyses were performed as previously described.

Net primary production (NPP), dark respiration (R), gross primary production (GPP), and light and dark calcification (G) rates were corrected from controls and calculated as Equations (1), (2), and (3), respectively:

(1) $$\text{NPP}\;\left(\text{or}\;R\right)=\frac{\Delta O_{2}\times V}{\Delta t\times \text{DW}}$$

(2) GPP=NPP+R

(3) $$G=-\frac{\Delta T_{A}\times V}{2\times \Delta t\times \text{DW}}$$

The ammonium flux was calculated according to the following equation:

(4) $${\text{NH}}_{4}^{+}=\frac{\Delta {\text{NH}}_{4}^{+}\times v}{\Delta t\times \text{DW}}$$

where ΔO_2_ and ΔT_A_ are respectively the differences between initial and final O_2_ concentrations (μmol O_2_ L^−1^) and T_A_ (μEq/L), *V* is the volume of the chamber (L), Δ*t* is the incubation time (hr), and DW is the dry weight of the algae (g).

In the absence of inhibition of the net primary production and calcification by high irradiances, NPP and G versus irradiance (*E*, μmol photons m^−2^ s^−1^) curve parameters were obtained according to Platt, Gallegos, and Harrison (1980):

(5) $$\text{NPP}={\text{GPP}}_{\max}\times \left(1-e^{\frac{-E}{\text{Ek}}}\right)-R$$

(6) $$G=G_{\max}\times \left(1-e^{\frac{-E}{\text{Ek}}}\right)-G_{D}$$

In the presence of inhibition of the net primary production and calcification by high irradiances, NPP‐E and G‐E curves were established by fitting Eilers and Peeters (1988) equation:

(7) $$\text{NPP}=\frac{I}{I^{2}\times a+I\times b+c}-R$$

(8) $$G=\frac{I}{I^{2}\times a+I\times b+c}-G_{D}$$

in Equations (5) and (7), *R* is the dark respiration rate; in Equations (6) and (8), G_D_ is the calcification rate in the dark. GPP_max_ and *G*_max_ are the maximum rates of gross primary production and calcification (μmol O_2_ or CaCO_3_ g DW hr^−1^), respectively, and *E*_k_ (μmol photons m^−2^ s^−1^) is the saturating irradiance.

In the presence of inhibition of the net primary production and calcification by high irradiances GPP_max_, *G*_max_, and *E*_k_ were calculated from the parameters *a*, *b*, and *c* in Equations (7) and (8) as:

(9) $${\text{GPP}}_{\max}\;\text{or}\;G_{\max}=\frac{1}{b+2\sqrt{\mathrm{ac}}}$$

(10) $$E_{k}=\frac{c}{b+2\sqrt{\mathrm{ac}}}$$

### 2.6 Dry weight and CaCO_3_ content in algae

After incubations, DW was measured on lyophilized maerl samples. The relative CaCO_3_ content (%) was calculated from the ash weight after burning at 550°C (5 hr) and the DW of samples (g CaCO_3_ g^−1^ DW maerl).

### 2.7 Chlorophyll a content

Maerl samples for chlorophyll *a* analyses were stored at −80°C prior to lyophilization. Afterward, they were ground in plastic tubes with 0.5 cm stainless steel beads (Brammer) using a Tissue Lyser II (QIAGEN). Extraction was done according to Arar and Collins (1997), that is, 5 ml of 90% acetone were added to 0.05 g of maerl powder. Samples were kept in glass tubes for 12 hr in dark and 4°C. Afterward, incubation tubes were centrifuged at 3,000 *g* for 10 min, and the supernatant was then transferred to a new tube; fluorescence was then measured using a calibrated Turner 10‐AU fluorometer.

### 2.8 Statistics

Statistical analyses were performed using the open source software R version 3.5.1 (R Core Team, 2017). The chlorophyll *a* and CaCO_3_ contents, and the metabolic rates were averaged for the two sets of thalli of each maerl species per aquarium, and the individual aquaria were thus considered as replicates (*n* = 3). Because of the small number of replicates per treatment, the normality of data was not verified for any of the analyses. This prevented us from using parametric analyses such as ANOVA or PERMANOVA and led us to use nonparametric analyses. Moreover, the use of rank‐based tests has been recommended for small samples (Legendre & Legendre, 1998) and is often as powerful as parametric tests (Scherrer, 2007). However, we are aware that such an approach increases the number of comparisons, and as such, there is a risk of a type I error, that is, incorrect rejection of the null hypothesis.

A two‐way nonparametric ANOVA (the *Scheirer*‐*Ray*‐Hare (SRH) test) was then performed to test the effects of the temperature–pH scenarios, nutrient availability, and their interaction on chlorophyll *a* and CaCO_3_ content, and primary production, respiration, and calcification rates under ambient irradiance and in the dark. NPP‐E and G‐E curve parameter fits were tested by the Fisher test using the following R script: *p*‐value = 1 − *pf* (*F*, *r*, *n*), where *F* is the Fisher test, *r* is the number of estimated parameters, and *n* is the number of points used to adjust the curve.

In both seasons, a nonparametric Kruskal–Wallis (KW) test was used to test differences in NPP‐E and G‐E curve parameters between present and future scenarios (Pr‐LN and Fu‐LN). For summer conditions, a two‐way nonparametric ANOVA (SRH test) was done to test the effects of the temperature–pH scenarios, nutrient availability, and their interaction on *L. corallioides* P‐E and G‐E curve parameters.

## 3 RESULTS

### 3.1 Physicochemical parameters

Salinity remained stable in both seasons at 35.0 ± 0.2. Mean values of seawater temperature, pH_T_, T_A_, DIC, *p*CO_2_, and Ω_Ar_ and mean nutrient concentrations for the four treatments and both seasons are given in Table 1.

**Table 1:** Mean values of temperature and carbonate system parameters and nutrient concentrations in each treatment (present (Pr) and future (Fu) scenarios with low (LN) or high nutrients (HN)) in both seasons

Treatment	*T* (°C)	pH_T_	T_A_ (μmol/kg SW)	DIC (μmol/kg SW)	*p*CO_2_ (μatm)	Ω_Ar_	PO_4_^3−^ (μmol/L)	NO_3_^−^ (μmol/L)	NH_4_^+^ (μmol/L)
Winter									
Pr‐LN	10.0 ± 0.1	8.04 ± 0.01	2,402 ± 18	2,213 ± 15	418 ± 4	2.14 ± 0.04	0.2–0.4	4.0–10.0	0.3–1.3
Pr‐HN	10.0 ± 0.1	8.04 ± 0.01	2,399 ± 15	2,209 ± 9	420 ± 8	2.10 ± 0.03	0.8–1.2	30.0–50.0	0.3–3.1
Fu‐LN	12.7 ± 0.2	7.71 ± 0.01	2,372 ± 16	2,298 ± 14	965 ± 9	1.16 ± 0.04	0.2–0.4	4.0–10.0	0.3–1.3
Fu‐HN	12.7 ± 0.2	7.71 ± 0.01	2,395 ± 24	2,318 ± 22	965 ± 10	1.14 ± 0.04	0.8–1.2	30.0–50.0	0.3–3.1
Summer									
Pr‐LN	18.0 ± 0.1	8.00 ± 0.01	2,375 ± 10	2,141 ± 11	450 ± 10	2.64 ± 0.02	0.3–0.4	3.2–6.4	0.2–0.5
Pr‐HN	18.0 ± 0.1	8.00 ± 0.01	2,351 ± 9	2,126 ± 20	464 ± 10	2.53 ± 0.08	0.9–1.7	25.0–55.0	0.2–0.5
Fu‐LN	20.7 ± 0.2	7.67 ± 0.01	2,414 ± 12	2,323 ± 12	1,187 ± 20	1.39 ± 0.03	0.3–0.4	3.2–6.4	0.2–0.5
Fu‐HN	20.7 ± 0.2	7.67 ± 0.01	2,424 ± 7	2,310 ± 16	1,182 ± 36	1.45 ± 0.04	0.9–1.7	25.0–55.0	0.2–0.5

Note

Temperature and pH_T_ were monitored every day in each aquarium (*n* = 90). Total alkalinity (T_A_) was measured each week (*n* = 20). Nutrients (PO_4_^3−^, NO_3_^−^ and NH_4_^+^) were measured twice per month (*n* = 7). Dissolved inorganic carbon (DIC), *p*CO_2_, and Ω_Ar_ were calculated from temperature, pH_T_, T_A_, and salinity (35) using the CO_2SYS_ software.

Abbreviation: SW, seawater.

### 3.2 Impact of the future temperature–pH scenario and nutrient enrichment in winter conditions under ambient irradiance

**Figure 3:**
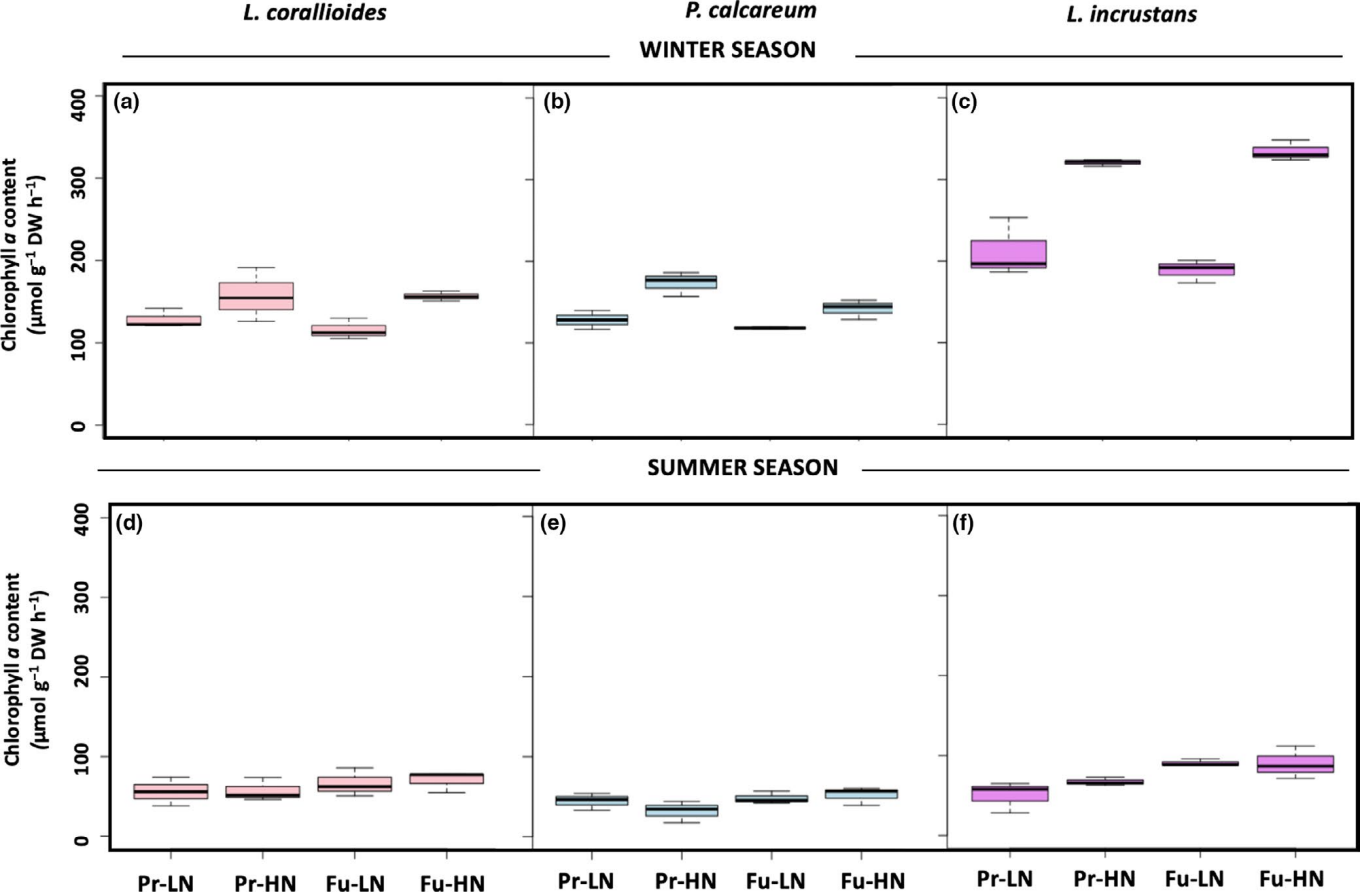
Box plots of the chlorophyll *a* content in *Lithothamnion corallioides*, *Phymatolithon calcareum*, and *Lithophyllum incrustans* in winter (a, b, c) and in summer (d, e, f) (*n* = 3) in the present (Pr) and future (Fu) scenarios with low (LN) or high nutrients (HN). Boxes extend from the 25th to the 75th percentiles of all the data for each treatment; the central horizontal line represents the median, and bars extend to the 95% confidence limits

Chl *a* content was significantly and positively affected by nutrient enrichment in the three maerl species (Appendix S1 & Figure 3a–c). CaCO_3_ content was not significantly affected by any treatment in none of the species (Appendix S1). Mean values were 85.3 ± 0.0% in *L. corallioides*, 85.7 ± 0.0% in *P. calcareum*, and 83.8 ± 0.0% in *L. incrustans*.

**Figure 4:**
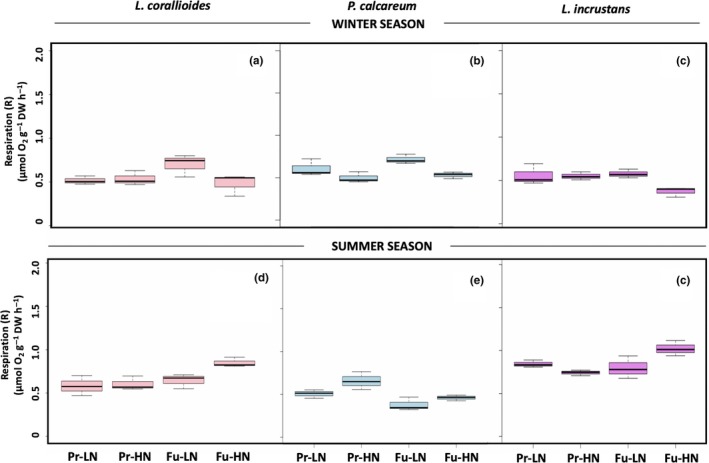
Box plots presenting respiration rates of *Lithothamnion corallioides*, *Phymatolithon calcareum*, and *Lithophyllum incrustans* in the winter (a, b, c) and in the summer (d, e, f) (*n* = 3) in the present (Pr) and future (Fu) scenarios with low (LN) or high nutrients (HN). Boxes extend from the 25th to the 75th percentiles of all the data for each treatment; the central horizontal line represents the median, and bars extend to the 95% confidence limits

**Figure 5:**
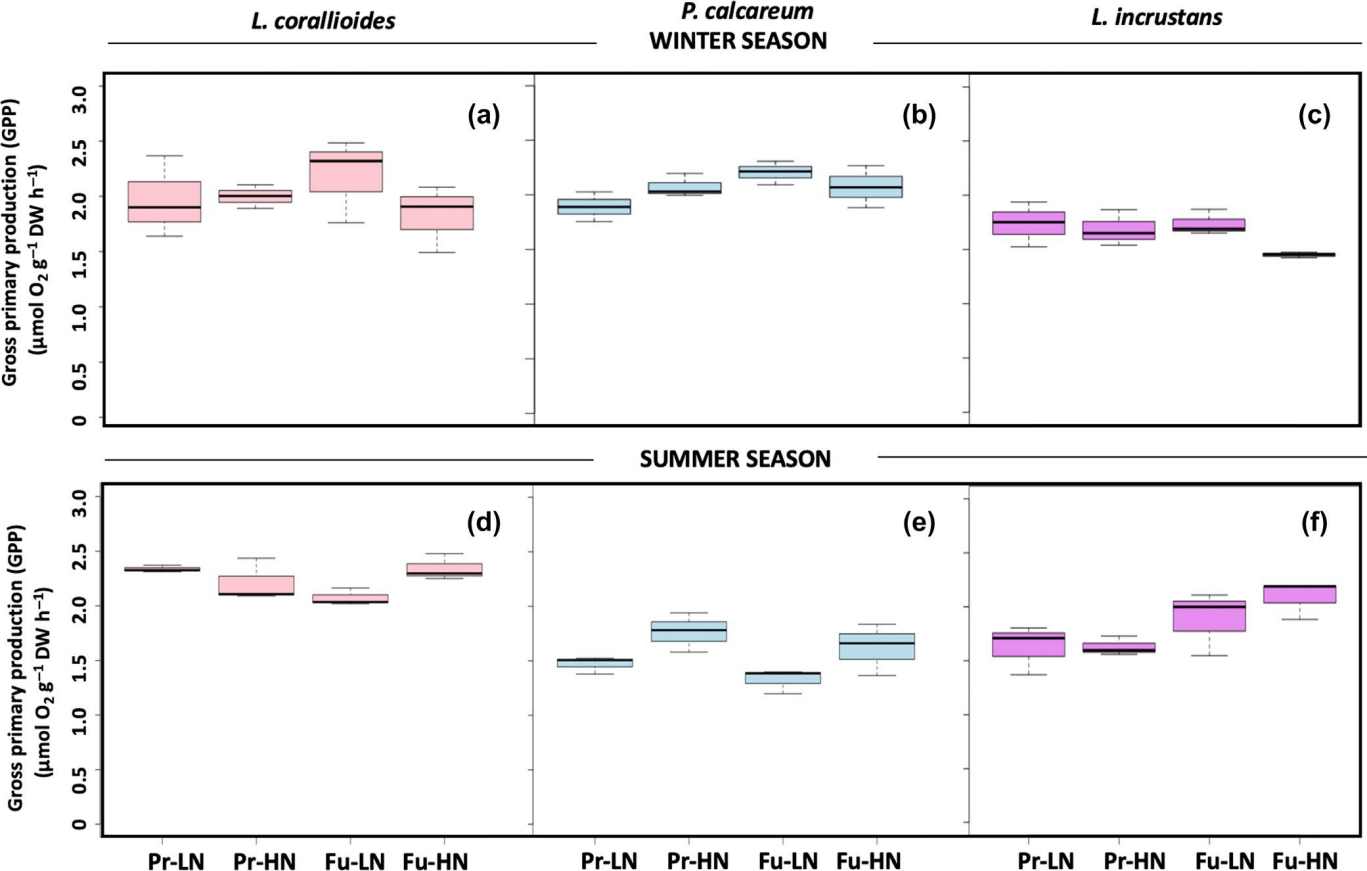
Box plots presenting gross primary production rates of *Lithothamnion corallioides*, *Phymatolithon calcareum*, and *Lithophyllum incrustans* in the winter (a, b, c) and in the summer (d, e, f) (*n* = 3) in the present (Pr) and future (Fu) scenarios with low (LN) or high nutrients (HN). Boxes extend from the 25th to the 75th percentiles of all the data for each treatment; the central horizontal line represents the median, and bars extend to the 95% confidence limits

**Figure 6:**
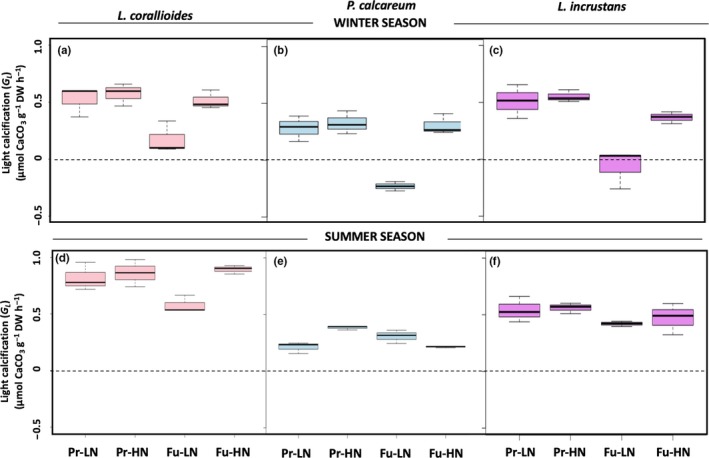
Box plots presenting net calcification rates in the light (G_L_) of *Lithothamnion corallioides*, *Phymatolithon calcareum*, and *Lithophyllum incrustans* in the winter (a, b, c) and in the summer (d, e, f) (*n* = 3) in the present (Pr) and future (Fu) scenarios with low (LN) or high nutrients (HN). Boxes extend from the 25th to the 75th percentiles of all the data for each treatment; the central horizontal line represents the median, and bars extend to the 95% confidence limits

*R* rates in *L. corallioides and L. incrustans* were not significantly affected by the temperature–pH scenario or nutrient conditions (Table 2 and Figure 4a,c). *R* rates in *P. calcareum* were negatively affected by nutrient enrichment (Table 2 and Figure 4b). GPP rates did not vary significantly across treatments or species (Table 2 and Figure 5c). G_L_ rates did not differ significantly between treatments in *L. corallioides*, although they were more than two‐fold lower under Fu‐LN relative to the other treatments (Table 2 and Figure 6a). *P. calcareum* showed net dissolution under Fu‐LN, but the test did not reveal any significant differences (Table 2 and Figure 6b). In *L. incrustans*, G_L_ rates were significantly affected by the temperature–pH scenario with net dissolution observed under Fu‐LN.

**Table 2:** Summary of the results of two‐way nonparametric (Scheirer–Ray–Hare) tests testing the effects of temperature and pH scenarios, nutrient enrichment and their interaction on *Lithothamnion corallioides*, *Phymatolithon calcareum*, *Lithophyllum incrustans* gross primary production (GPP), respiration (R), light (G_L_) and dark calcification (G_D_) rates, and G_L_:GPP ratio in winter and summer conditions (*n* = 3)

	*df*	Gross primary production (GPP) (μmol O_2_ g^−1^ DW hr^−1^)		Respiration (R) (μmol O_2_ g^−1^ DW hr^−1^)		Calcification in the light (G_L_) (μmol CaCO_3_ g^−1^ DW hr^−1^)		Calcification in the dark (G_D_) (μmol CaCO_3_ g^−1^ DW hr^−1^)		G_L_:GPP ratio	
		*F*	*p*‐value	*F*	*p*‐value	*F*	*p*‐value	*F*	*p*‐value	*F*	*p*‐value
*Lithothamnion corallioides/*WINTER											
Scenario	1	0.02	.873	0.92	.337	2.56	.109	8.31	.004**	0.92	.337
Nutrients	1	0.41	.522	1.64	.200	2.56	.109	0.92	.337	2.08	.149
Interaction	1	0.92	.337	2.08	.149	1.26	.262	0.23	.631	2.56	.109
*Phymatolithon calcareum/*WINTER											
Scenario	1	3.10	.078	0.92	.337	2.08	.149	5.77	.016*	2.56	.109
Nutrients	1	0.00	1.000	5.02	.025*	3.10	.078	0.23	.631	2.08	.149
Interaction	1	2.56	.109	3.00	.631	1.26	.262	2.08	.149	1.64	.200
*Lithophyllum incrustans/*WINTER											
Scenario	1	1.26	.262	0.92	.337	6.56	.010*	0.03	.873	6.56	.010*
Nutrients	1	3.69	.055	2.08	.149	1.26	.262	2.56	.109	1.64	.200
Interaction	1	1.64	.200	3.69	.055	0.41	.522	2.56	.109	0.23	.631
*Lithothamnion corallioides/*SUMMER											
Scenario	1	0.92	.337	3.69	.055	1.26	.262	2.08	.149	1.64	.200
Nutrients	1	0.41	.522	1.26	.262	3.69	.055	0.03	.873	3.69	.055
Interaction	1	4.33	.037*	1.64	.200	1.64	.200	3.10	.078	0.64	.423
*Phymatolithon calcareum/*SUMMER											
Scenario	1	1.26	.262	5.77	.016*	1.26	.262	6.56	.010*	0.03	.873
Nutrients	1	4.33	.037*	2.08	.149	0.23	.631	0.00	1.000	0.23	.631
Interaction	1	0.03	.872	0.23	.631	7.41	.006**	3.10	.078	7.41	.006**
*Lithophyllum incrustans/*SUMMER											
Scenario	1	4.33	.037*	2.08	.149	3.69	.055	0.02	.873	6.56	.010*
Nutrient	1	0.64	.423	0.23	.631	0.64	.423	6.56	.010*	0.03	.873
Interaction	1	0.64	.423	5.77	.016*	0.10	.749	0.10	.749	0.00	1.000
*Lithothamnion corallioides*											
Season	1	4.81	.028*	4.08	.043*	12.81	<.001***	0.96	.326	8.67	.003**
*Phymatolithon calcareum*											
Season	1	14.52	<.001***	3.85	.050	0.16	.686	0.01	.908	4.81	.028*
*Lithophyllum incrustans*											
Season	1	2.43	.119	16.80	<.001***	17.28	<.001***	12.40	.001**	0.48	.488

Note

Comparisons among seasons were done using a one‐way nonparametric (Kruskal–Wallis) test. Analyses significant at the *α* = .025 level are indicated by asterisks.

Values in bold refer to parameters that are significantly different.

**Figure 7:**
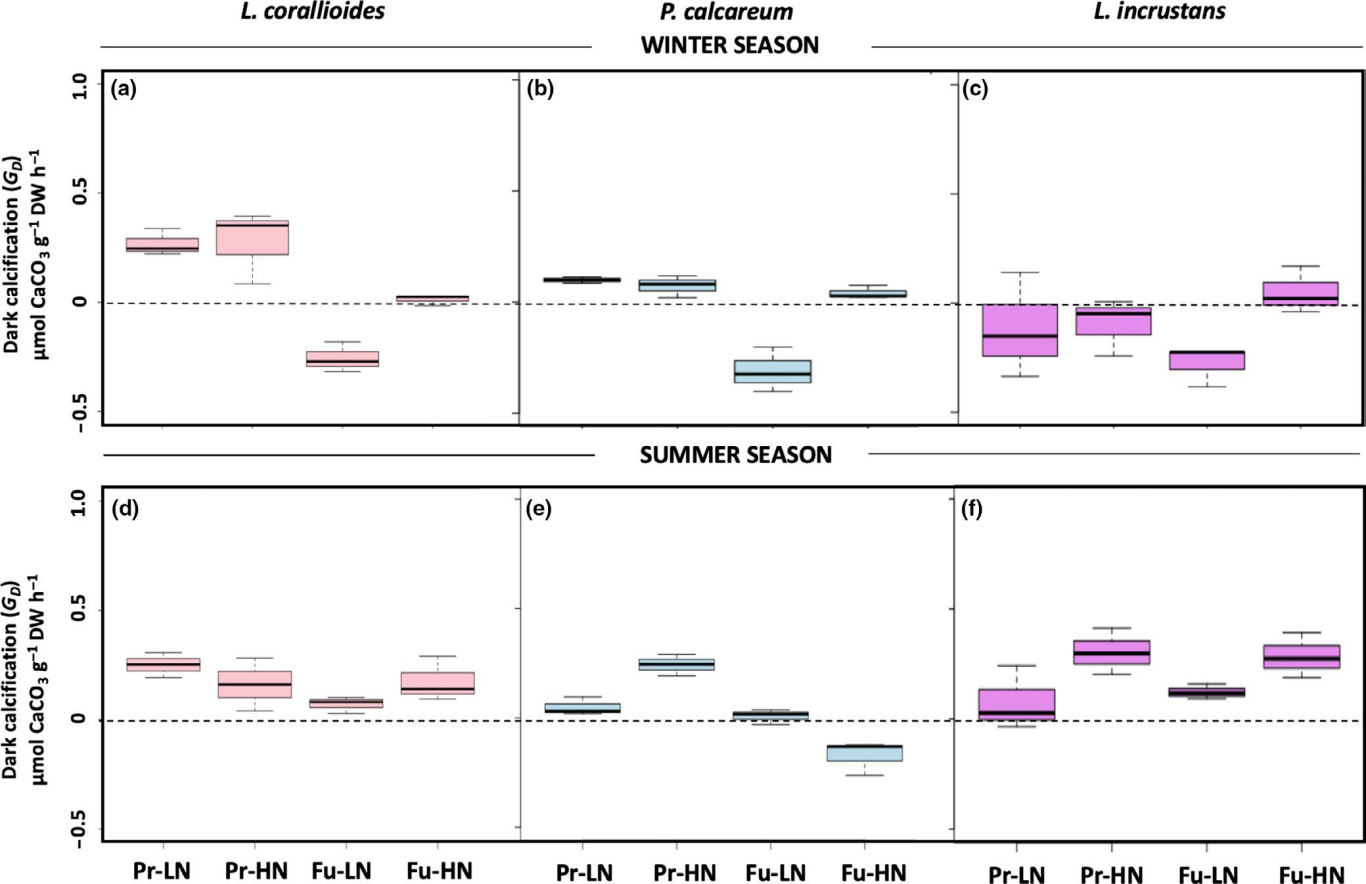
Boxes presenting net calcification in the dark (G_D_) of *Lithothamnion corallioides*, *Phymatolithon calcareum*, and *Lithophyllum incrustans* in the winter (a, b, c) and in the summer (d, e, f) (*n* = 3) in the present (Pr) and future (Fu) scenarios with low (LN) or high nutrients (HN). Boxes extend from the 25th to the 75th percentiles of all the data for each treatment; the central horizontal line represents the median, and bars extend to the 95% confidence limits

*Lithothamnion corallioides* and *P. calcareum* winter G_D_ rates were negatively affected by the future temperature–pH scenario (Table 2), and net dissolution was observed under Fu‐LN (Figure 7a,b). *Lithophyllum incrustans* G_D_ rates did not differ significantly between treatments (Table 2), although dissolution was observed under Fu‐LN (Figure 7c).

G_L_:GPP ratio did not vary significantly between treatments in *L. corallioides* (0.23 ± 0.11) and *P. calcareum* (0.09 ± 0.19) (Table 2). In *L. incrustans*, it ranged from −0.03 ± 0.10 (Fu‐LN) to 0.33 ± 0.00 (Pr‐HN) and was negatively affected by the future temperature–pH scenario (Table 2).

### 3.3 Impact of the future temperature–pH scenario and nutrient enrichment in summer conditions under ambient irradiance

*Lithothamnion corallioides* and *P. calcareum* Chl *a* content was affected neither by the temperature–pH scenario nor by nutrient conditions (Figure 3d,e) but, in *L. incrustans*, Chl *a* was positively affected by the future temperature–pH scenario (Appendix S1 & Figure 3f). CaCO_3_ content ranged from 84.6 (Fu‐LN) to 86.4% (Pr‐LN) in *L. corallioides* with significant lower values under the future temperature–pH scenarios. In *P. calcareum*, it ranged from 85.0% (Pr‐LN) to 86.5% (Fu‐LN) and was affected by the interaction between scenario and nutrients. It was not significantly affected by any treatment in *L. incrustans* and averaged 84.4 ± 0.0%.

*Lithothamnion corallioides R* rates were not affected by any treatment; in *P. calcareum*, *R* was negatively affected by the temperature–pH scenario. In *L. incrustans*, *R* rates were significantly affected by the interaction between the temperature–pH scenario and nutrient availability (Table 2 & Figure 4d–f). GPP of *L. corallioides* was also affected by the interaction between the temperature–pH scenario and nutrient availability (Table 2 & Figure 5d). GPP rates increased with nutrient enrichment in *P. calcareum* (Table 2 & Figure 5e) and with the future temperature–pH scenario in *L. incrustans* (Table 2 & Figure 5f). None of the species observed dissolution in the light in the summer. *Lithothamnion corallioides* G_L_ was not significantly affected by any of the treatments, although the G_L_ rate was 39% lower under Fu‐LN than under Fu‐HN (Table 2 & Figure 6d). In *P. calcareum*, G_L_ was affected by the interaction between the temperature–pH scenarios and nutrient availability (Table 2 & Figure 6e): when nutrients were added, it increased under the present scenario and decreased under the future scenario. In *L. incrustans*, G_L_ rates were affected neither by the temperature–pH scenario nor by nutrient enrichment (Table 2 & Figure 6f). Calcification was observed in the dark in *L. corallioides* under all treatments, and no effect was observed (Table 2 & Figure 7d). As in winter conditions, *P. calcareum* G_D_ rates were negatively affected by the temperature–pH scenario (Table 2) with net dissolution observed under Fu‐HN (Figure 7e). *Lithophyllum incrustans* G_D_ was significantly affected by nutrient availability (Table 2): It was higher under HN conditions than LN conditions (Figure 7f).

The *L. corallioides* G_L_:GPP ratio did not differ significantly between treatments and was on average of 0.35 ± 0.06. The *P. calcareum* summer G_L_:GPP ratio ranged from 0.13 ± 0.02 (Fu‐HN) to 0.23 ± 0.05 (Fu‐LN) and was significantly affected by the interaction between the temperature–pH scenario and nutrient availability (Table 2). In *L. incrustans*, the summer G_L_:GPP ratio was significantly lower under the future temperature–pH scenario (mean value of 0.22 ± 0.04) than under present scenarios (mean value of 0.34 ± 0.06).

### 3.4 Impact of the future temperature–pH scenario on the relationship between net primary production/calcification and irradiance in winter conditions

**Figure 8:**
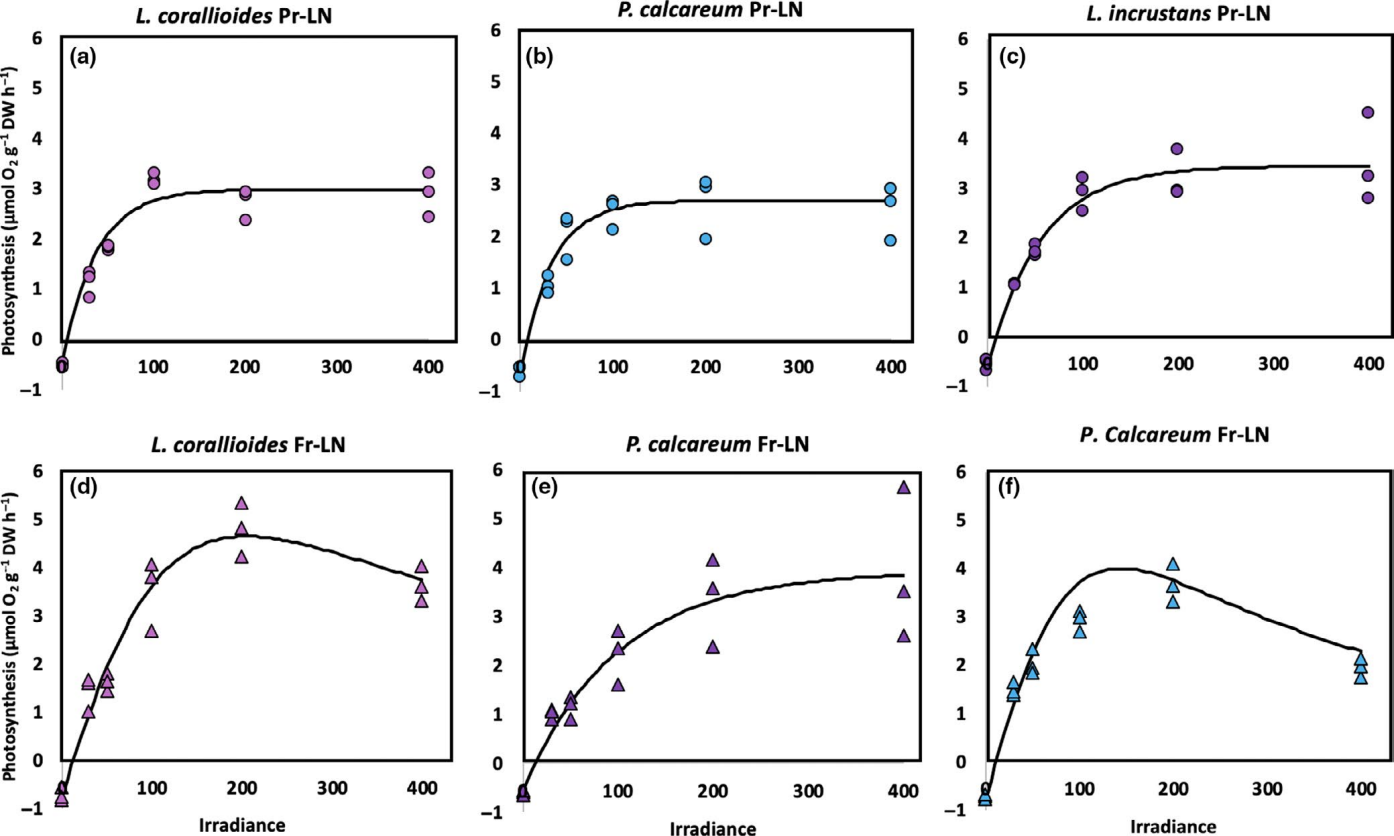
Relationship between net primary production rates and irradiance in *Lithothamnion corallioides*, *Phymatolithon calcareum*, and *Lithophyllum incrustans* under unenriched (LN) conditions in the present temperature–pH scenario (Pr‐LN, a, b, c, respectively) and in the future temperature–pH scenario (Fu‐LN, d, e, f, respectively) in winter conditions

**Figure 9:**
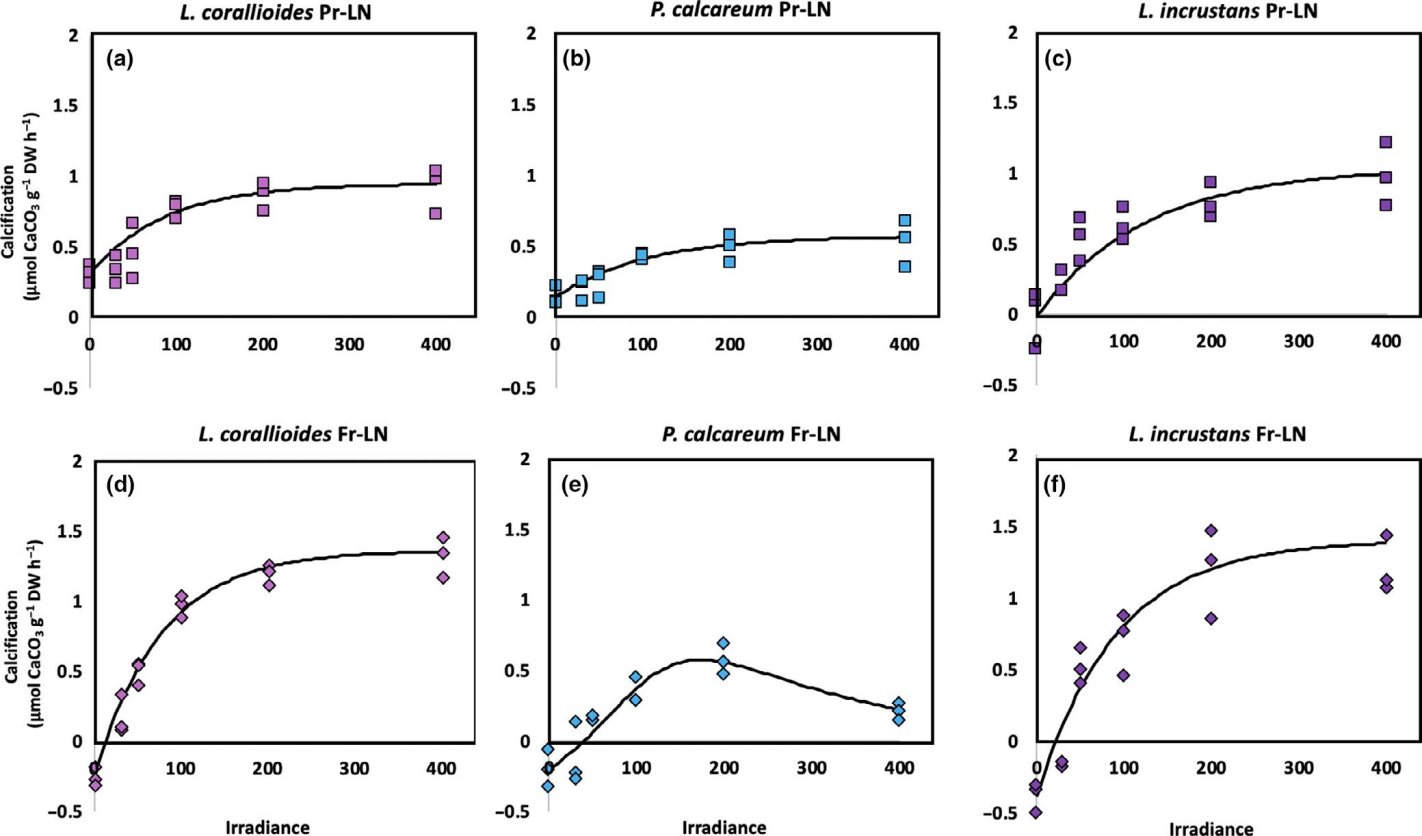
Relationship between net calcification rates and irradiance in *Lithothamnion corallioides* under unenriched (LN) conditions in the present temperature–pH scenario (Pr‐LN, a, b, c, respectively) and in the future temperature–pH scenario (Fu‐LN, d, e, f, respectively) in winter conditions

**Figure 10:**
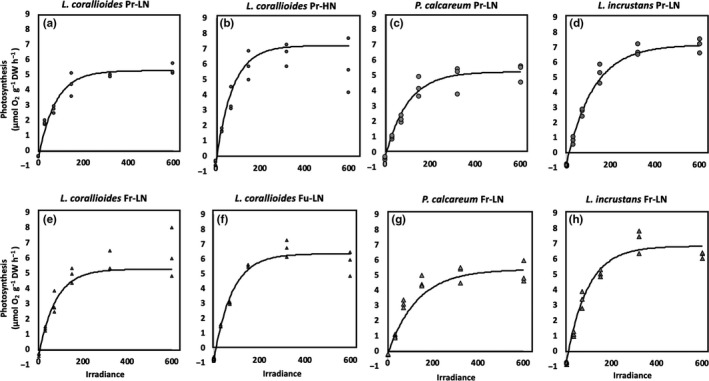
Relationship between net primary production rates and irradiance in *Lithothamnion corallioides* under unenriched (LN) and enriched conditions (HN), *Phymatolithon calcareum* and *Lithophyllum incrustans* under unenriched conditions in the present temperature–pH scenario (Pr, a, b, c, d, respectively) and in the future temperature–pH scenario (Fu, e, f, g, h, respectively) in summer conditions

The curves of the relationship between winter net primary production (NPP) or calcification (G) and irradiance (E) are shown on Figures 8 and 9 (*R* ^2^ > 0.90). GPP_max_ increased significantly in the future temperature–pH scenario in *L. corallioides* and *P. calcareum*. Both species showed photoinhibition at this season at irradiances above 200 μmol photons m^−2^ s^−1^ (Figure 8). Photosynthetic irradiance of saturation (*E*_k_) was enhanced under the future scenario in *L. corallioides* (Figure 8 & Table 3). In the three maerl species, *G*_max_ was enhanced under the future scenario (Figure 9, Tables 3 and 4). In *P. calcareum*, inhibition was observed at irradiances above 200 μmol photons/m^2^ hr^−1^ (Figure 10). Calcification *E*_k_ was only affected in *L. corallioides*, where it was higher under the future scenario (Figure 9; Tables 3 and 4).

**Table 3:** Summary of the results of one‐way nonparametric (Kruskal–Wallis) tests on the effects of temperature–pH scenarios under the unenriched nutrient conditions in *Lithothamnion corallioides*, *Phymatolithon calcareum*, *Lithophyllum incrustans* on the parameters of the relationships between primary production (NPP) or calcification (*G*) and irradiance (*E*) in winter and summer conditions (*n* = 3)

Species	*df*	Primary production				Calcification			
		GPP_max_		*E*_k_		GPP_max_		*E*_k_	
		*F*	*p*	*F*	*p*	*F*	*p*	*F*	*p*
*Lithothamnion corallioides/*WINTER	1	3.86	.049*	3.86	.049*	3.86	.049*	0.43	.513
*Phymatolithon calcareum/*WINTER	1	3.97	.046*	0.44	.507	3.86	.049*	0.05	.827
*Lithophyllum incrustans/*WINTER	1	0.05	.827	2.33	.127	3.86	.049*	0.43	.513
*Lithothamnion corallioides/*SUMMER	1	3.86	.049*	1.19	.275	3.86	.049*	3.86	.049*
*Phymatolithon calcareum/*SUMMER	1	0.43	.513	2.33	.127	0.05	.827	2.33	.127
*Lithophyllum incrustans/*SUMMER	1	2.33	.126	3.23	.072	0.43	.513	2.33	.127
*Lithothamnion corallioides/SEASON*	1	6.56	.010*	0.64	.423	0.92	.337	1.26	.262
*Phymatolithon calcareum/SEASON*	1	5.79	.016*	8.34	.004**	0.00	1.000	1.26	.262
*Lithophyllum incrustans/SEASON*	1	8.31	.004**	3.12	.077	3.10	.078	2.56	.109

Note

Comparison of the season effect on the parameters of the NPP‐E and G‐E curves was done using a nonparametric test (Kruskal–Wallis) (*n* = 3). GPP_max_, the maximal gross primary production (μmol O_2_ g^−1^ DW hr^−1^), *E*_k_, the irradiance of saturation (μmol photons m^−2^ s^−1^), *G*_max_, the maximal gross calcification (μmol CaCO_3_ g^−1^ DW hr^−1^). Analyses significant at the *α* = .025 level are indicated by asterisks.

Values in bold refer to parameters that are significantly different.

**Table 4:** Mean parameters of the relationship between net primary production (NPP) or calcification (*G*) rates and irradiance under the present temperature–pH scenario (Pr‐LN) and future scenario (Fu‐LN) under winter and summer conditions (*n* = 3)

Species	Net primary production (μmol O_2_ g^−1^ DW hr^−1^)		Calcification (μmol CaCO_3_ g^−1^ DW hr^−1^)	
	GPP_max_	*E*_k_	*G*_max_	*E*_k_
*Lithothamnion corallioides/*WINTER				
Pr‐LN	3.5 ± 0.2	37 ± 11	0.64 ± 0.14	87 ± 40
Fu‐LN	5.2 ± 0.7	99 ± 13	1.62 ± 0.16	77 ± 8
*Phymatolithon calcareum/*WINTER				
Pr‐LN	3.3 ± 0.4	34 ± 14	0.43 ± 0.15	104 ± 35
Fu‐LN	4.6 ± 0.0	47 ± 0	0.87 ± 0.16	133 ± 45
*Lithophyllum incrustans/*WINTER				
Pr‐LN	4.0 ± 0.9	56 ± 16	1.05 ± 0.25	125 ± 90
Fu‐LN	4.5 ± 1.8	99 ± 47	1.78 ± 0.41	91 ± 17
Pr‐LN	5.7 ± 0.2	76 ± 14	1.6 ± 0.0	95 ± 27
*Lithothamnion corallioides/*SUMMER				
Pr‐HN	7.7 ± 0.4	75 ± 12	1.3 ± 0.2	97 ± 70
Fu‐LN	7.2 ± 1.0	105 ± 34	1.0 ± 0.1	46 ± 5
Fu‐HN	7.1 ± 0.4	79 ± 6	1.2 ± 0.2	55 ± 10
*Phymatolithon calcareum/*SUMMER				
Pr‐LN	5.6 ± 1.0	99 ± 20	0.65 ± 0.03	129 ± 63
Fu‐LN	5.6 ± 0.2	81 ± 15	0.65 ± 0.13	71 ± 31
*Lithophyllum incrustans/*SUMMER				
Pr‐LN	8.0 ± 0.2	120 ± 9	2.27 ± 1.30	122 ± 23
Fu‐LN	7.6 ± 0.4	93 ± 16	2.28 ± 0.07	161 ± 23

Note

GPP_max_,the maximal gross primary production rates (μmol O_2_ g^−1^ DW hr^−1^), *E*_k_, the irradiance of saturation (μmol photon m^−2^ s^−1^), *G*_max_, the maximal gross calcification rates (μmol CaCO_3_ g^−1^ DW hr^−1^).

### 3.5 Impact of the future temperature–pH scenario on the relationship between net primary production/calcification and irradiance in summer conditions

**Figure 11:**
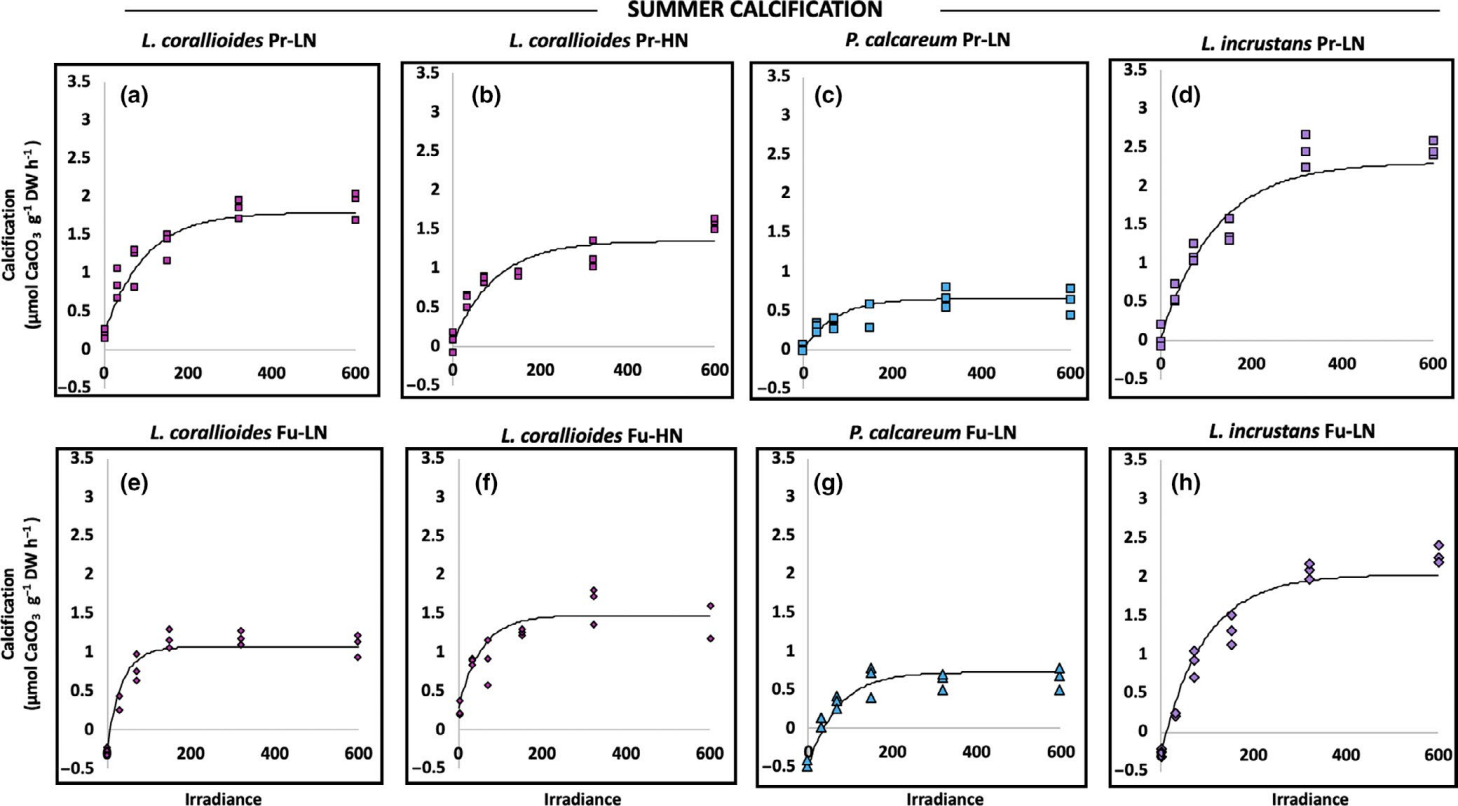
Relationship between net calcification rates and irradiance in *Lithothamnion corallioides* under unenriched (LN) and enriched conditions (HN), *Phymatolithon calcareum*, and *Lithophyllum incrustans* under unenriched (LN) conditions in the present temperature–pH scenario (Pr, a, b, c, d, respectively) and in the future temperature–pH scenario (Fu, e, f, g, h, respectively) in summer conditions

The curves of the relationship between NPP or G and E are shown in Figures 10 and 11 (*R* ^2^ > 0.90). Only *L. corallioides* GPP_max_ increased significantly under the future scenario (Tables 3 and 4). Photosynthetic irradiance of saturation (*E*_k_) did not vary significantly among treatments in any of the species (Figure 10 & Table 4). *Lithothamnion corallioides G*_max_ dropped significantly (of 37%) under the future scenario, but did not vary significantly between temperature–pH scenarios in the other species (Figure 11; Tables 3 and 4). Calcification irradiance of saturation (*E*_k_) did not vary among treatments in any of the species (Figure 11; Tables 3 and 4).

### 3.6 NH_4_^+^ consumption

NH_4_^+^ consumption did not vary significantly between species and treatments whatever the season (ANOVA, *p* > .05), but winter consumption was significantly affected by irradiance (ANOVA, *F* = 96.06, *p* < .001). The highest value was observed at 400 μmol photons m^−2^ s^−1^ (0.06 ± 0.02 μmol NH_4_^+^ g^−1^ DW hr^−1^) and the lowest at 30 μmol photons m^−2^ s^−1^ (0.006 ± 0.01 μmol NH_4_^+^ g^−1^ DW s^−1^). The NH_4_^+^ summer consumption did not vary with irradiance (ANOVA, *p* > .05), but was higher (0.07 ± 0.02 μmol g^−1^ DW s^−1^) than in winter (ANOVA, *p* < .001).

## 4 DISCUSSION

### 4.1 Effect of projected global climate change on physiological parameters

The response of the three studied maerl species to future pH and temperature scenarios varied with the season. This is in agreement with previous studies on coralline algae reporting that the effects of ocean warming and acidification are modified by seasonal variation in species responses and environmental conditions (Martin, Cohu, et al., 2013; Martin & Hall‐Spencer, 2017). In temperate ecosystems, maerl beds face strong seasonal variability of physicochemical parameters (Qui‐Minet et al., 2018) including variations in temperature and irradiance (length of photoperiod and intensity). Rising sea temperature can be beneficial in winter for coralline algae, allowing an increase in their photosynthetic and calcification rates (Martin, Cohu, et al., 2013). However, increased temperature in summer above the range of temperatures experienced in natural habitats could be detrimental for coralline algae; moreover, as it could be aggravated by decreased pH (Martin & Gattuso, 2009). In addition, illumination factors such as length of photoperiod and intensity affect the physiology of coralline algae (Martin, Castets, & Clavier, 2006; Martin, Charnoz, et al., 2013; Martin et al., 2007; Martin, Cohu, et al., 2013). The length of the photoperiod directly influences diel patterns of respiration and net photosynthesis and their coupling to calcification. In the light, the photosynthetic fixation of CO_2_ causes an increase in the pH within the intracellular spaces and at the boundary layer of diffusion that induces CaCO_3_ precipitation (Cornwall, Hepburn, Pilditch, & Hurd, 2013; De Beer & Larkum, 2001). Conversely, in the dark, the respiration process generates CO_2_, resulting in a decrease of pH within the intracellular spaces and the boundary layer of diffusion (Cornwall et al., 2013; Hurd, Harrison, Bischof, & Lobban, 2014) and thus hindering calcification.

In future climate change scenarios, the three maerl species may thus be particularly sensitive during the winter period when photoperiod is short. Indeed, under this scenario, calcification rates in winter significantly decreased in *L. incrustans*, while dissolution was observed in *P. calcareum* under ambient irradiance (30 μmol photons m^−2^ s^−1^). By contrast, the lack of effect of the future scenario in summer under ambient irradiance suggests a positive effect of long photoperiods and the ability of the three species to cope with lower values of pH under elevated temperatures.

Light intensity has an important role in the response of coralline algae to climate change. For example, in winter, maximum rates of gross primary production under high irradiances were enhanced under the future scenario in *L. corallioides* and *P. calcareum*, while the rates of gross primary production under ambient irradiance were unaffected. This may reflect the absence of dissolved inorganic carbon (DIC) limitation under ambient irradiance in comparison with higher irradiances. It is thus of particular importance to determine photosynthetic rates as a function of irradiance since light availability can affect the ability of algae to use DIC. For example, DIC limitation can be influenced by irradiance as carbon concentrating mechanisms are energy dependent (Raven, Giordano, Beardall, & Maberly,^2012^).

Mechanisms that control light harvesting and photoprotection depend on light intensity while they may be controlled by temperature and pH variations (Croce & Van Amerongen, 2014). Therefore, due to seasonal changes in irradiance, the outcome of light intensity interaction with global climate change will directly depend on the season. *Lithothamnion corallioides* and *P. calcareum* presented a certain degree of inhibition of *net primary production* and *net calcification* under high irradiances, while no photoinhibition was observed for *L. incrustans*. The maerl species *L. corallioides* and *P. calcareum* possess zeaxanthin as a photoprotective pigment, whereas *L. incrustans* possess lutein as a photoprotective pigment and has a significantly higher concentration of pigments relative to the other species (Qui‐Minet et al., 2018). Therefore, although the three studied maerl species can be found at the same location in our study site, they have different geographical distribution, reflecting different ecological affinities. Indeed, the response to the future climate change scenario of maerl is here dependent on the species. Species‐specific responses to global climate change under different irradiances may result from differences in morphology and taxonomical groups, that may be translated in differences in light reflectance, type, and concentration of photoprotective pigments (Burdett et al., 2014; Vazquez‐Elizondo & Enriquez, 2016).

In this work, we studied how the combination of ocean acidification and warming impacted the photosynthetic and calcification response of maerl species to different irradiances. Maximum rates of gross primary production and calcification rates are known to increase with increasing temperature up to a thermal optimum and then decline steeply with further increase in temperature (Chalker & Taylor, 1975; Eilers & Peeters, 1988; Hurd et al., 2014; Marshall & Clode, 2002). Those parameters were not enhanced by the future summer scenario in the here studied maerl species, *L corallioides*. Therefore, we assume that *P. calcareum* and *L. incrustans* photosynthetic and calcification rates may decline with further rise in temperature. Furthermore, the significant decrease in the maximum rate of calcification under high irradiances in *L. corallioides*, and its significant lower CaCO_3_ content at this season under the future summer scenario suggests a significant decrease of CaCO_3_ precipitation at low tide at this season, when irradiances are >300 μmol photons m^−2^ s^−1^.

Knowledge of the environmental conditions at the location where the species were collected is essential to understand how they may be impacted by global climate change. Likewise, the maerl bed (Roz bed located in the Bay of Brest), where the three maerl species were collected has a shallow depth (*chart datum* 0.7 m) and faces a high variability of light intensity resulting from cloud cover, turbidity, and tide variation. According to our results, the three maerl species will be more vulnerable to dissolution and/or their calcification rates will decrease in the near future during the winter season due to low light intensities (30 μmol photons m^−2^ s^−1^). Although, in the summer, the photoperiod and light intensity may increase; summer epiphyte algal species show high coverage of the Roz maerl bed, which severely decreases the light intensity that maerl receives. However, in this study, photosynthesis and calcification were not impacted negatively by none of the scenarios at low irradiances in the summer.

Species‐specific consequences of ocean warming and acidification are also noticed for the respiration of the maerl species. The respiration rates of *L. corallioides* and *L. incrustans* were not affected by the future pH and temperature scenario either in winter or in summer, while those of *P. calcareum* decreased significantly under the future summer scenario. Compared to the two other maerl species, *P. calcareum* has a higher affinity for lower temperatures (Adey & McKibbin, 1970) and may be more sensitive to increased temperature in summer. The lack of effect of decreased pH and increased temperatures on respiration rates in *L. corallioides* and *L. incrustans* may point to an adaptation to the near future conditions of ocean acidification and warming due to the substantial seasonal and diel pH and temperature variability experienced in their natural environment in the Roz maerl bed (Qui‐Minet et al., 2018) although this was not the case for *P. calcareum*, living in the same place.

Considering our experimental system, a tank effect due to potential contamination within a header tank (spread of a disease or pollution) cannot be completely discarded and would have biased the results due to the interdependence of replicates. Nevertheless, due to the high seawater renewal rate and frequency of cleaning of the header tanks, such a risk of contamination was limited. Furthermore, our results are in agreement with what has been observed in situ at the Bay of Brest, in terms of the primary production and calcification seasonal tendency (Martin et al., 2006) and species‐specific responses (Z. N. Qui‐Minet, D. Davoult, J. Grall, C. Delaunay, C. Six, T. Cariou, S. Martin, unpublished).

### 4.2 Effect of nutrient enrichment on physiological parameters

Coastal ecosystems are impacted by local changes, nutrient enrichment being one of the most relevant (Aufdenkampe et al., 2011). Species‐specific responses were observed. *Lithothamnion corallioides* was not impacted by nutrient availability under any treatment and season. The significant decrease of *P. calcareum* respiration resulting from nutrient enrichment under winter season may be a way to minimize carbon dioxide losses and promote calcification, although the former process was not significantly affected at this season. Interestingly, in the summer an increase of nutrient availability impacted positively the rates of calcification in the dark in *L. incrustans* and gross primary production under ambient light irradiance in *P. calcareum*.

The absence of negative effects of nutrient enrichment on calcification contradicts previous studies that have described phosphate as a crystal poison due to its negative impact on the calcification rates of coralline algae (Bjork et al., 1995; Kinsey & Davies, 1979; Simkiss, 1964), even at the concentrations reported in the present study (1 μmol/L; Bjork et al., 1995).

Physical factors (e.g., light intensity, photoperiod, and temperature) influence nutrient uptake kinetics in macroalgae (Fogg, 1953; Harrison & Hurd, 2001; Hofmann, Straub, & Bischof, 2013; Hurd et al., 2014; Magnusson, Larsson, & Axelsson, 1996; Ravaglioli et al., 2017). A strong correlation between seawater N concentration, algal N content, and algal pigment content has been observed for temperate macroalgae (Gevaert et al., 2001; Davison, Jordan, Fegley, & Grobe, 2007; Chow, 2012; Bordeyne, Migné, & Davoult, 2016), including the maerl species studied here (Qui‐Minet et al., 2018). The increase in Chl *a* content under nutrient‐enriched conditions in winter suggests that the uptake of N was not saturated under the treatment without enrichment (Hurd et al., 2014). Thus, high nutrient concentrations in winter might have a positive impact on temperate coralline algae physiology. Conversely, the lack of a nutrient loading effect on the summer chlorophyll *a* content of the three maerl species may indicate that, during this season, maerl have higher nutrient requirements and therefore would not store N.

### 4.3 Interaction between global climate change and local change on physiological parameters

Environmental variations of natural and anthropogenic origin, including nutrient availability, may increase coralline algae resilience or exacerbate their response to global climate change (Williamson et al., 2014). According to some authors, the response of calcified organisms to the future conditions of temperature and pH will directly depend on nutrient availability (Celis‐Plá et al., 2015; Langdon & Atkinson, 2005). Nevertheless, its impact has not been previously studied in maerl/rhodolith species. Several authors reported a negative effect of phosphate on the calcification rates of coralline algae (Bjork et al., 1995; Kinsey & Davies, 1979; Simkiss, 1964). However, higher phosphate concentrations (1 μmol/L) did not decrease the calcification rates in any of the species under projected future temperature–pH conditions (winter and summer) at ambient irradiance and at dark.

Although nonsignificant, the mean calcification rates of *L. corallioides* under a projected future winter scenario under ambient irradiance were about 50% higher under nutrient‐enriched conditions relative to unenriched conditions. Moreover, net calcification remains positive (precipitation > dissolution) for the three maerl species under nutrient‐enriched conditions (under ambient irradiance and in dark); whereas, net dissolution is observed in both light and dark conditions under unenriched conditions. In summer, none of the species showed net dissolution independently of nutrient availability.

These results are consistent with previous observations on calcifying organisms suggesting that energy supply helps them to cope with higher *p*CO_2_ (Schoepf et al., 2013; Tanaka et al., 2013; Ramajo et al., 2016). Nevertheless, the ratio between the rates of G_L_ and gross primary production for *P. calcareum* decreased significantly under the future pH/temperature scenario and nutrient‐enriched conditions. Nutrient availability can modify the enzymatic activity of algae and therefore the energetic balance and the response of photosynthesis and calcification processes to elevated CO_2_ and increased temperature (Hofmann et al., 2013; Williamson, Perkins, Voller, Yallop, & Brodie, 2017). However, to our knowledge, there is no information regarding the energy allocation of maerl in an ocean acidification and warming context.

Since pigments are N‐containing components (Hurd et al., 2014), maerl specimens observing inhibition of winter gross primary production and net calcification under high irradiances were probably under deficient conditions of nitrogen.

The relationship between primary production/calcification and irradiance under different nutrient concentrations was studied only in *L. corallioides* in summer, and we did not observe any interactive effect between scenario and nutrients on the maximal rates of gross primary production and calcification (GPP_max_ and *G*_max_). The nonresponsiveness to these conditions may suggest the nonlimitation of nutrients for this species, likely due to the capacity of macroalgae to stock N during previous seasons and to their low energetic requirements (Hurd et al., 2014). Further studies should consider the response and potential interaction between global climate change and nutrient loading on the light harvesting and calcification mechanisms of coralline algae, in order to give a deeper insight into their capacity to adapt to future scenarios. These results suggest that despite any possible artifact introduced by the experimental system, nutrient enrichment can modify the response of the algae under a given scenario.

## 5 CONCLUSION

The magnitude of the ongoing global climate change effects on temperate maerl species located in coastal systems will depend on how their fitness responds to it in interaction with seasonal variations of temperature, photoperiod, light intensity, and nutrient availability, among other physicochemical parameters. It will also depend on the interaction with other stressors or sources of local changes, such as freshwater inputs, hydrodynamics, pollution, and among others (Horta et al., 2016; Qui‐Minet et al., 2018).

Although care must be taken in the interpretation of the results due to potential pitfalls related to experimental design, our results suggest:

- An antagonistic interaction between global climate change and nutrient enrichment with nutrient loading ameliorating some of the negative effects of global climate change.
- The ability of ocean acidification and warming to impair light harvesting and photoprotective mechanisms of maerl algae, while this may depend on the interaction with other abiotic factors.
- Species‐specific responses to global climate change suggest that dominance and species distribution may deeply change in the future (Brodie et al., 2014). For instance, although species can adapt to the same environment, they are sensitive to specific thresholds of environmental parameters. Our study suggests that ocean acidification and warming would render some species more vulnerable to stressful conditions of irradiance than others. In this context, *P. calcareum* when living at shallow depths (thus, subject to higher light intensities) may be severely affected by global climate change relative to shallow adapted species such *L. incrustans*. *P. calcareum* is also the species with the lowest calcification rates and the only one to endure dissolution in the dark under the future summer scenario, which may threaten its growth rates and survival in shallow depths and lower latitudes.

Given these results, we expect that temperate maerl species will benefit from moderate nutrient enrichment in the winter season, avoiding dissolution under ambient low irradiance levels. Being *L. corallioides* the most abundant species in the Bay of Brest, the significant decrease of its calcification rates in the summer will have a negative impact on the CaCO_3_ budget in this location. Furthermore, previous studies have shown a positive impact of ocean acidification on the development of fleshy epiphytic macroalgae (Johnson, Price, & Smith, 2014). Many of the species developing on North Atlantic maerl beds are consider as opportunists (Bunker, Brodie, Maggs, & Bunker, 2017) and may also benefit from the increase of temperature and nutrient availability with negative consequences for maerl survival. In this context, fleshy epiphytic macroalgae compete with maerl for light, carbon, and nutrients (Steneck, 1986). They can also modify the seawater chemistry within the diffusive boundary layer and increase the diel pH fluctuations under future global change scenarios (Short, Pedersen, & Kendrick, 2015). However, epiphytic abundance is also affected by other abiotic and biotic parameters such as hydrodynamics (Hily, Potin, & Flocíh, 1992), the antifouling ability of coralline algae (Figueiredo, Norton, & Kain, 1997) and grazing by the benthic fauna (Guillou, Grall, & Connan, 2002). Furthermore, coastal ecosystems are highly heterogeneous, and maerl beds located in these ecosystems are subject to different sources of local change which affect their resilience and capacity to adapt to future scenarios of global change (Horta et al., 2016). Due to their slow growth rates (Potin, Floc'h, Augris, & Cabioch, 1990), activities such as dredging may reduce their abundance at a higher rate than global climate change (Grall & Hall‐Spencer, 2003; Hall‐spencer, Grall, Moore, & Atkinson, 2003).

We are aware that the small number of replicates resulting from a constraining experimental design prevented us from carrying out a more powerful single multifactorial analysis. This weakness is a consequence of the process of testing and answering a complex but realistic problem, the combined action of climate change and local eutrophication in a multifactorial experimental design. Therefore, the scope of our study was limited, and our results should be considered carefully. Despite interdependence of replicates per scenario, this paper contains useful information to increase our ability to predict the future effects of ocean acidification.

Such experiment could have been improved by increasing the number of header tanks to avoid pseudo‐replication as recommended by Cornwall and Hurd (2016). However, the number of tank and aquarium units would multiply rapidly in such an experiment, manipulating both climate change scenarios and nutrient concentrations at multiple levels. This would have been prohibitive.

Further studies considering other abiotic parameters and biotic interactions need to be carried in order to develop management policies capable to increase the resilience of maerl beds under the future global climate change scenario.

## CONFLICT OF INTEREST

None declared.

## AUTHOR CONTRIBUTIONS

Z.N.Q.M., S.M., D.D., and J.G. designed research; Z.N.Q.M., J.C., and S.M. performed research; Z.N.Q.M, M.M.‐S., and T.C. contributed samples; Z.N.Q.M. and D.D. analyzed data; Z.N.Q.M., S.M, D.D., and J.G. wrote the paper.

## Data Availability

All data supporting this study are provided as a Dryad file: https://doi.org/10.5061/dryad.zkh18935j
