# Correction to “Combined effects of global climate change and nutrient enrichment on the physiology of three temperate maerl species”

**DOI:** 10.1002/ece3.72607

**Published:** 2026-02-23

Qui‐Minet ZN, Coudret J, Davoult D, et al. 2019. Combined effects of global climate change and nutrient enrichment on the physiology of three temperate maerl species. Ecology and Evolution 9, 13787–13807. https://doi.org/10.1002/ece3.5802.

The species names in Figure 1 are not presented in the correct order in the published article. The correct figure caption is shown below:

**FIGURE 1:**
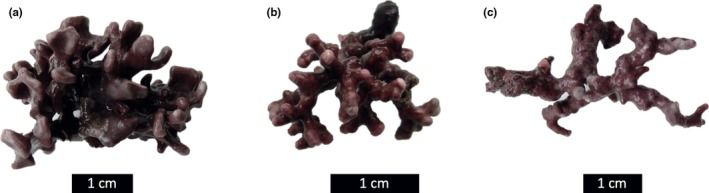
Specimens of (a) *Lithophyllum incrustans*, (b) *Lithothamnion corallioides*, and (c) *Phymatolithon calcareum* collected in the Roz maerl bed in the Bay of Brest (Brittany, France) (photos Coralie Delaunay). Scale bars = 1 cm.

We apologize for this error.

