# Supplementary material for: Combined effects of global climate change and nutrient enrichment on the physiology of three temperate maerl species
Source: Ecol Evol. 2019 Dec 5;9(24):13787–807. doi: 10.1002/ece3.5802 (PMC6953553; doi:10.1002/ece3.5802)
Supplement: Supplementary file 1 [file ECE3-9-13787-s001.docx]

**APPENDIX S1**

Summary of results of the the two-way non-parametric (Scheirer-Ray-Hare) tests testing the effects of the temperature-pH scenario, nutrient enrichment, and their interaction in *L. corallioides*, *P. calcareum*, and *L. incrustans* Chlorophyll *a* and CaCO_3_ contents in winter and summer conditions (n=3). Comparison among seasons was done using a one-way non-parametric (Kruskal-Wallis) test.

|  |  |  | **Chlorophyll *a*** | | **CaCO_3_** | |
| --- | --- | --- | --- | --- | --- | --- |
|  |  |  |  | |  | |
|  |  | df | F | p-value | F | p-value |
| *L. corallioides/* | Scenario | 1 | 0.02 | 0.873 | 0.10 | 0.749 |
| WINTER | Nutrients | 1 | 6.56 | **0.010*** | 3.69 | 0.055 |
|  | Interaction | 1 | 0.41 | 0.522 | 2.08 | 0.149 |
| *P. calcareum/* | Scenario | 1 | 1.26 | 0.262 | 0.92 | 0.337 |
| WINTER | Nutrients | 1 | 7.41 | **0.006**** | 1.26 | 0.262 |
|  | Interaction | 1 | 0.23 | 0.631 | 0.41 | 0.522 |
| *L. incrustans/* | Scenario | 1 | 0.16 | 0.688 | 3.69 | 0.055 |
| WINTER | Nutrients | 1 | 8.34 | **0.004**** | 0.41 | 0.522 |
|  | Interaction | 1 | 0.78 | 0.378 | 0.26 | 0.262 |
| *L. corallioides/* | Scenario | 1 | 2.08 | 0.149 | 8.31 | **0.004**** |
| SUMMER | Nutrients | 1 | 0.03 | 0.873 | 0.00 | 1.000 |
|  | Interaction | 1 | 0.23 | 0.631 | 0.15 | 0.149 |
| *P. calcareum/* | Scenario | 1 | 2.56 | 0.109 | 0.03 | 0.873 |
| SUMMER | Nutrients | 1 | 0.10 | 0.749 | 0.41 | 0.522 |
|  | Interaction | 1 | 1.26 | 0.262 | 8.31 | **0.004**** |
| *L. incrustans/* | Scenario | 1 | 7.41 | **0.006**** | 0.00 | 1.00 |
| SUMMER | Nutrients | 1 | 0.10 | 0.749 | 0.23 | 0.631 |
|  | Interaction |  | 0.92 | 0.337 | 2.56 | 0.109 |
| *L. corallioides* | Season | 1 | 17.28 | **< 0.001***** | 0.96 | 0.326 |
| *P. calcareum* | Season | 1 | 17.28 | **< 0.001***** | 0.01 | 0.908 |
| *L. incrustans* | Season | 1 | 17.29 | **< 0.001***** | 6.45 | **0.011*** |
